# Clinico-Metabolic Profile and Follow-Up of Familial Cases Compared to Sporadic Cases in a Lyon Series of Type 1 Diabetic Children

**DOI:** 10.7759/cureus.60080

**Published:** 2024-05-11

**Authors:** Nabila Chekhlabi, Marc Nicolino, Kévin Perge

**Affiliations:** 1 Pediatric Departement, Cheikh Khalifa International University Hospital, Mohammed VI University of Health Sciences (UM6SS), Casablanca, MAR; 2 Pediatric Endocrinology, Diabetology, and Metabolism Department, L'hôpital Femme Mère Enfant, Hospices Civils de Lyon, Claude Bernard University, Lyon, FRA

**Keywords:** metabolic profile, paternal transmission, type 1 diabetes, sporadic t1d, familial t1d

## Abstract

Objective: This study aimed to describe the clinical, biochemical, therapeutic, and progressive characteristics of children with familial type 1 diabetes (T1D) compared to those with non-familial T1D. Compare within the first group, the phenotype of type 1 diabetics inherited from the father with those inherited from the mother.

Patients and methods: We conducted a retrospective study lasting 10 years at the L'hôpital Femme Mère Enfant (Woman-Mother-Child Hospital) in Lyon, France. Cases were any child diagnosed with T1D for at least 12 months who had a parent with T1D. Each case was matched with a T1D control without a family history of T1D, of the same age, same sex and same year of discovery. Cases group was divided into two subgroups according to the sex of the parent with T1D.

Results: A total of 43 children had a TD1 parent (family group) of whom 27 cases were the father. Forty four T1D children without any T1D parent were matched (sporadic group). The family group had consulted earlier (p < 0.001), were less in initial diabetic ketoacidosis (p = 0.016), and had a lower HbA1C level lower (p < 0.001) and lower initial insulin requirements (p < 0.001). During follow-up, it was noted that the evolution of Hb1AC, insulin requirements, and chronic complications were similar in familial and non-familial cases (p = 0.943, p = 0.450, p = 0.664, respectively). The patients in the T1D mother group seemed better balanced than those of the T1D father with an average HbA1C at 10 years of follow-up of 7.82% in the maternal group compared to 9.10% in the paternal group (p = 0.021).

Conclusion: This study shows that familial T1D is a protective factor against the initial severity of T1D in offspring. Paternal T1D presents a more severe initial and progressive clinico-biological character than T1D inherited from the mother. However, during follow-up, other psycho-environmental factors could modify this observation.

## Introduction

Type 1 diabetes (T1D) is responsible for hyperglycemia secondary to insulin deficiency resulting from the autoimmune destruction of pancreatic β cells [1]. It is well-established that patients with T1D have a genetic predisposition [1]. However, several elements suggest that this genetic predisposition cannot, on its own, explain the pathogenesis of T1D: sharp annual increase in the incidence of T1D over the last 30 years, significant geographical disparities in incidence, and modification of risk of T1D in migrants according to their country of residence and discordance in prevalence (50-70%) in monozygotic twins [2]. Thus, it is now accepted that environmental factors largely participate in the penetrance of T1D in a genetically predisposed population. The genetic contribution had been amply suggested by the relatively high degree of multiplex families with T1D. Indeed, approximately 10-15% of patients with T1D have relatives affected by T1D, whether parents or siblings [3,4]. To date, more than 80 T1D predisposition genes or loci have been identified.

Surprisingly, Warram and colleagues were among the first (in 1984) to report a higher risk of T1D in children of fathers with T1D compared to children of affected mothers with a risk calculated at 6.1 ± 1.8 and 1.3 ± 0.9%, respectively [5]. Other studies have confirmed this increased risk of transmission of T1D from the father compared to the mother [6-10]. Concerning the initial presentation, while there is a higher rate of diabetic ketoacidosis at the discovery of T1D in people with sporadic T1D compared to familial T1D, the initial clinico-biological picture is more serious in the offspring of a father affected by T1D compared to the offspring of an affected mother [3,9,11,12]. All these elements suggest that paternal T1D seems to be more frequent and more severe with the hypothesis of a higher risk of long-term diabetic complications compared to maternal T1D [13]. Studies of this phenomenon, however, are limited.

The objective of our study is to compare the clinical, metabolic, therapeutic, and evolutionary profiles of T1D patients whose parents are T1D with those of non-diabetic families. The secondary objective is to compare the profiles of T1D patients of paternal origin with those of maternal origin.

## Materials and methods

We conducted a retrospective observational and analytical study carried out in the pediatric diabetology and endocrinology department of L'hôpital Femme Mère Enfant (Woman-Mother-Child Hospital), Hospices Civils de Lyon, Lyon, France. Cases of familial diabetes and sporadic diabetes were selected over a 10-year period from January 2011 to December 2020. Data were collected from the computerized files of the pediatric diabetology service and consultation.

The inclusion criteria for the familial group were any child or adolescent under 18 years of age and diagnosed with type 1 diabetes for at least 12 months, followed by the department's diabetology doctors and having a T1D father or mother. The diagnosis of T1D is based on clinical symptoms suggesting diabetes mellitus with positive autoantibodies and/or evocative HLA marking. Newly diagnosed diabetics (less than 12 months) were excluded due to difficulty in judging the progression of the disease. Other types of diabetics (type 2 diabetes, cystic fibrosis diabetes, or monogenic diabetes) and those lost to follow-up or patients followed by another medical structure were also excluded. Each selected familial case was matched with a type 1 diabetic control of the same age, sex, and year of discovery. A ratio of 1:1 was applied.

Thus, group 1 represented children and adolescents with T1D whose father or mother has T1D, while group 2 contained diabetic patients whose two parents were free of T1D. Group 1 was also divided into two subgroups according to the sex of the parent with T1D (father (group 1a) and mother (group 1b)).

The initial clinical presentation and metabolic assessment of T1D (blood pH, C-peptide, and Hb1Ac), prevalence of β-cell autoimmunity (anti-glutamic acid decarboxylase (GAD) antibodies, anti-islet cell antibodies, anti-insulin, and zinc transporter 8 (ZnT8)), thyroid autoimmunity (anti-thyroglobulin antibodies and anti-thyroid peroxidase (TPO)), and anti-trans-glutaminases type IgA during discovery were recorded and analyzes. Insulin therapy (pump or basal/bolus regimen, initial insulin dose, and carbohydrate counting) and the evolving profile (body mass index, insulin requirement over time, occurrence of acute complications and chronic conditions, and the evolution of Hb1Ac) were statistically compared between the different groups and subgroups.

Statistical methods

Data were coded and entered using Jamovi statistical software version 1.6.23. Data were summarized using mean, standard deviation, median, minimum, and maximum for quantitative variables, frequencies, and percentages for categorical or qualitative variables. Qualitative variables were analyzed using the chi-square test or Fisher's exact test in the event of insufficient theoretical numbers. Quantitative variables were analyzed by Student's t-test or one-way ANOVA with Tukey's post-hoc test for parametric variables and Mann-Whitney U test or Kruskal-Wallis test with post hoc test of Dunn for non-parametric variables. The association analysis between certain parameters is measured by Spearman correlation analysis. The significance level was set at 5% and p-values less than 0.05 were considered statistically significant. Univariate analyses were performed for each model and variables significantly increasing the probability were loaded into the respective multivariate models.

## Results

Among a total of 1,004 diabetic children followed by the department between 2011 and 2020, we identified a T1D father in 27 (2.7%) of our patients and a T1D mother in 16 (1.6%). A total of 43 children (group 1) presented with T1D and had a T1D parent, of whom 27 (62.8%) were the father and 16 (37.2%) were the mother. Forty-four type 1 diabetic children without any T1D parents were matched (group 2). The demographic, clinico-biological, and therapeutic characteristics of the discovery of T1D of cases and controls are presented in Table 1. Indicative of good matching, the median age of the children and the sex ratio were identical between the two groups. Likewise, the two sexes also had the same distribution within group 1 (P = 0.847) (Table 1). 

**Table 1:** Comparison of demographic, clinico-biological, and therapeutic characteristics at the discovery of T1D between familial and sporadic T1D cases (n (%)/mean ± SD). * X2, ** Student's t-test, *** Mann-Whitney, BMI: body mass index

	Group 1 parents DT1 (N = 43)	Group 2 parents not DT1 (N = 44)	P-value
Age n (%)			
<5 years	20 (46.5%)	20 (45.4%)	0.950*
5–10 years	11 (25.5%)	11 (25%)	0.921*
10–15 years	8 (18.6%)	9 (20.4%)	0.828*
>15 years	4 (9.4%)	4 (9.1%)	0.973*
Sex boy/girl	25/18	26/18	0.926*
Normal BMI at admission n (%)	32 (74.4%)	29 (65.9%)	0.111*
Diagnosis time n (%)			
<8 days	25 (58.1%)	5 (11.3%)	< 0.001*
8–15 days	14 (32.6%)	12 (27.3%)	0.075*
>15 days	4 (9.3%)	27 (61.4%)	< 0.001*
Diabetic ketoacidosis at admission n (%)	6 (13.9%)	16 (36.3%)	0.016*
Hb1Ac at admission (Mean)	9.6	11.4	< 0.001**
Peptide C µg/l (median)	0.3 (0.2-0.5)	0.2 (0.2-0,4)	0.127***
Insuline dose U/kg/d (Mean)	0.60 +/- 0.379	0.95 +/- 0.404	< 0.001**
Autoantibody positivity rate n (%)	41 (95.3%)	38 (86.4%)	0.147*
Insulin therapy			
Pump n (%)	11 (25,5%)	12 (27,3%)	0.858*
Multi-injection regimen n (%)	32 (74.5%)	32 (72.7%)	
Functional insulin therapy	18 (41.8%)	15 (34.1%)	0.455*

The comparison of the discovery of T1D, between the cases and the controls, showed a statistically significant difference concerning the time to diagnosis of diabetes (P < 0.001), the occurrence and severity of diabetic ketoacidosis (P = 0.016), the rate of Hb1AC (P < 0.001), and initial insulin requirement (P < 0.001). Familial T1D patients (group 1) were less frequently in initial diabetic ketoacidosis with earlier diagnosis and had a lower HbA1C level and initial insulin requirement levels. A positive and significant correlation was observed between the initial Hb1Ac level, and the insulin dose required on admission (Pearson coefficient P < 0.001, r = 0.578) and between the latter and the frequency of diabetic ketoacidosis at discovery (Pearson coefficient P = 0.001, r = 0.336).

There was no significant difference when comparing the initial body mass index (BMI) level at diagnosis (P = 0.111), the C peptide level (P = 0.127), the level of positive diabetic autoantibodies (P = 0.147), the type of therapy chosen (insulin pump or multi-injection regimen) (P = 0.858), and the frequency of use of carbohydrate counting (P = 0.455) (Table 1). 

Concerning the evolution of diabetes in the two groups (Table 2), the average duration of follow-up was similar between them with an average duration of 6.5 years +/- 3.80 for the case group and 6.6 years +/- 3.46 for the control group (P = 0.873). There is no statistically significant difference in the occurrence of acute complications (diabetic ketoacidosis P = 0.778 and severe hypoglycemia P = 0.147) or chronic microangiopathic complications (diabetic retinopathy P = 0.664 and nephropathy P = 0.295). The change in patients' BMI, Hb1AC level, and insulin requirement over time was similar among familial and non-familial cases (P = 0.559, P = 0.943, P = 0.450, respectively). The occurrence of autoimmune hypothyroidism was more frequent in the first familial T1D group (8 (18.6%) vs. 0 (0%), P = 0.003) (Table 2).

**Table 2:** Comparison of the clinico-biological evolution of the two groups (n (%)/mean ± SD). * X2, ** Fisher's exact test, *** Student's t-test

	Group 1	Group 2	P value
Follow-up duration (Mean +/- SD)	6.5 +/- 3.8	6.6 +/- 3.4	0.873**
Diabetic ketoacidosis n (%)	5 (11.6%)	6 (13.6%)	0.778*
Severe hypoglycemia n (%)	2 (4.6%)	7 (15.9%)	0.157**
Pump success n (%)	16 (37.2%)	16 (36.7%)	0.868*
Retinopathy n (%)	2 (4.6%)	3 (6.8%)	0.664*
Nephropathy n (%)	1 (2.3%)	3 (6.9%)	0.295*
Cœliac disease n (%)	6 (13.9%)	2 (4.5%)	0.129*
Autoimmune thyroiditis n (%)	8 (18.6%)	0 (0%)	0.003*
Normal BMI n (%)	32 (74.4%)	36 (81.8%)	0.632*
insulin requirement (Mean)	0.8 +/- 0.447	0.8 +/- 0.345	0.450***
Hb1Ac level at follow-up (mean)	7.8 +/- 0.97	8 +/- 1.01	0.418

**Figure 1:**
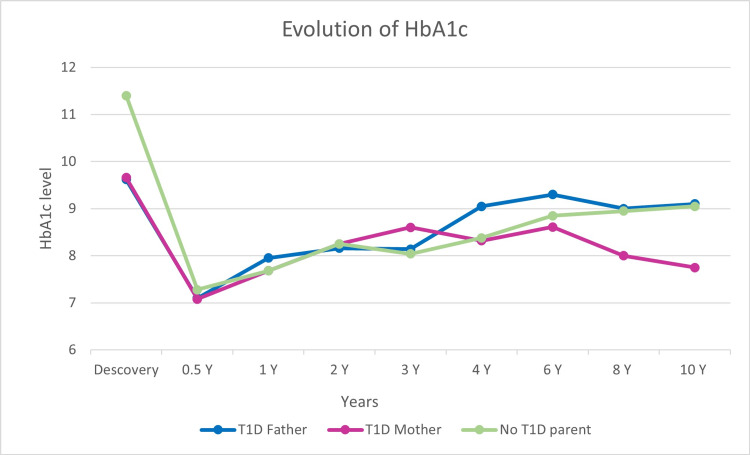
Evolution of the HbA1c level during follow-up.

The analysis and comparison of the characteristics of the two subgroups (T1D father and mother) did not note any significant difference in the age, sex, and initial BMI of the children. Baseline HbA1c and autoantibody levels were similar between the two subgroups (P = 0.954 and P = 0.265, respectively). The frequency of ketoacidosis at discovery was slightly higher in the 1a-father T1D group without reaching the level of statistical significance with a p at 0.265 (1a = 5 (18.5%) vs. 1b = 1 (6.2%)) (Table 3). The average insulin doses at discovery were slightly different with 0.56 IU/Kg/day for subgroup 1a and 0.65 IU/Kg/day in subgroup 1b. The patients in the 1b-mother T1D subgroup seemed better balanced than the 1a-father T1D subgroup with an average HbA1C at four years of follow-up of 8.3% in the 1b-mother T1D group compared to 9% in the 1a-father T1D group and after 10 years of evolution of 7.8% against 9.1% (P = 0.021) (Figure 1). Complications seem more frequent in the 1a-father T1D group compared to the 1b-mother T1D group but without a statistically significant difference. Conversely, autoimmune diseases seem more frequent in the 1b-T1D mother group (Table 3). 

**Table 3:** Comparison between the groups of children with T1D father and T1D mother. * AB: antibody, HBA1C: hemoglobin A1C

	Group 1a father DT1 (n = 27)	Group 1b mother DT1 (n = 16)	P-value
Mean age years	7.5	6.1	0.328
Ratio sex	1.45	1.28	0.847
Ketoacidosis at diagnosis n (%)	5 (18.5%)	1 (6.2%)	0.262
Initial hemoglobin A1C (Hb1AC)	9.6%	9.6%	0.998
Initial dose of insulin U/kg/day	0.5	0.6	0.949
Positivity of antibody n (%)	25 (92.6%)	16 (100%)	
1 AB*	7 (28%)	5 (31.3%)	0.760
2 AB	11 (44%)	8 (50%)	0.463
3 AB	7 (28%)	3 (18.7%)	0.546
Ketoacidosis at follow-up n (%)	2 (7.4%)	3 (18.7%)	0.262
Severe hypoglycemia n (%)	0 (0%)	2 (12.5%)	0.06
Hospitalization for severe imbalance n (%)	10 (37%)	5 (31.3%)	0.416
Microangiopathic complications n (%)	3 (11.1%)	1 (6.2%)	0.596
Autoimmune disease n (%)	8 (29.6%)	6 (37.5%)	0.594
Mean of HbA1c at 10 years	9.1%	7.8%	0.021

## Discussion

Familial T1D represents an average percentage of 10-12% of cases of T1D in children and adolescents [4]. In our study, we identified a T1D father in 27 (2.7%) of diabetic children followed in the service and a T1D mother in 16 (1.6%). Several studies carried out on Caucasian populations report that fathers with T1D transmit this disease to their offspring two to three times more frequently than their female counterparts. In a large study carried out in Sweden over a period of 15 years and comparing annually the proportion of patients with and without family members with T1D, the relative frequency of familial cases remains similar over the years [10]. The average annual percentage of all patients having a first-degree relative with insulin-dependent diabetes was 11.7% (range 9.2-16.7) [10]. The number of fathers with insulin-dependent diabetes was 2.5 times higher than the number of mothers [10]. In another US database containing 1244 families with 2,156 non-diabetic and diabetic offspring of parents with T1D, the 20-year risk of T1D in offspring of diabetic fathers and mothers was 8.9 ± 1.0 and 3.4 ± 0.6, respectively [14]. For mothers carrying T1D before or after age 8, the risk of T1D in offspring is 13.9 ± 4.4 and 2.4 ± 0.6% at age 20, respectively. Among Finns also, at the time of diagnosis, 4-7% of T1D children have a father with T1D while only 1.5 to 3% have an affected mother [3]. In one of the largest international cohorts, called EURODIAB ACE covering results from 18 countries, it was noted that a greater proportion of T1D fathers (3.4%) had children with T1D than mothers with T1D (1.8%), giving a relative risk of T1D of 1.8 (95% CI 1.4 to 2.5) [15]. This phenomenon has given rise to hypotheses about genetic and non-genetic factors, but the mechanism remains obscure.

Among the genetic and/or environmental theories generated to explain this preferential transmission, there is the well-established fact that men have a higher prevalence of T1D than women and an apparent difference in fertility between the two sexes affected by T1D with more offspring on the father's side (2.4 vs. 1.8 in the mother) and accordingly a significant birth order effect [16]. In a cohort called TRIGR following children genetically at risk of T1D for more than eight years, it was confirmed that the risk of developing beta cell autoantibodies was lower when the children were born to mothers with T1D than in those born to an affected father [17]. Genetically, no predisposition to T1D and no presence of a predisposing or protective allele has been mapped on either of the X or Y sex chromosomes to date [16]. Genetic imprinting, which is the differential expression of genetic material at the allelic level depending on whether it comes from the male or female parent, could be responsible for the decreased expression of diabetogenic genes in the offspring of diabetic mothers [14]. Julier et al. described an association between paternal meiotic events and transmission of an allele in region 5 of the insulin gene to HLA-DR4-positive diabetic offspring. In this study, the T1D-associated allele was transmitted to 38 of 50 HLA-DR4-positive diabetic offspring of male parents, while the effect of maternally transmitted alleles was not significant [18]. By contrast, another study done in the United Kingdom found different results. They found that the relationship between this region and HLA is not specifically DR4 dependent, but also DR3 dependent, and that there is maternal and paternal genetic distortion [14].

Another explanation could be related that increased exposure, during pregnancy and infancy, to insulin and proinsulin and anti-insulin antibodies produced by the mother (as a part of her disease and in response to insulin replacement therapy) in maternal offspring with T1D leads to mechanisms of central and peripheral tolerance to (pro)insulin, which is reflected in the reduction of CD4+ Ly T responses to proinsulin in cord blood and at nine months and therefore provides relative protection against T1D [16,19]. It has been reported that the INS genotype predisposing to T1D is linked to decreased thymic insulin expression and consequently an increased risk of developing anti-insulin autoantibodies and increased T cell responses to proinsulin. This supports the hypothesis that T1D or maternal hyperglycemia increases insulin expression in the neonatal thymus and therefore improves immune tolerance and provides some protection against T1D [20,21]. This immune tolerance during pregnancy could partly explain the selective transmission of T1D between diabetic fathers and mothers.

Maternal microchimerism, which is the transfer of maternal cells to the fetus during pregnancy, is also an avenue for understanding the familial transmission of T1D. Maternal cells transmitted to the fetus through the placenta are made mainly of hematopoietic stem cells capable of differentiating into different cells, particularly pancreatic cells [22]. Protection of fetal immune detection occurs through the differentiation of fetal T cells into regulatory T lymphocytes leading to immune tolerance. On the other hand, according to the same references, if these cells are in large quantities above a certain threshold, they will be an easy target for the immune attack and therefore become non-self [22].

As shown in our study and the literature, patients without type 1 diabetes in the family presented more severe metabolic decompensation at the time of diagnosis than those with a familial disease [4]. The paternal history of T1D was negatively associated with the presence of diabetic ketoacidosis in children [23]. The severity of the initial clinical picture and long-term complications seem more frequent in the T1D father group than in the T1D mother group without a statistically significant difference in our cohort. No clear explanation is described, but as Turtinen et al. noted in their publication, the T1D mother would be more present and more concerned by the first signs, by monitoring and care during the illness than the diabetic father [4].

The limitations of our study are its retrospective nature and the relatively small number of cases. Nevertheless, our data should be considered representative because they come from a regional center that follows more than 1000 diabetic children. Our study is one of the few in the literature to compare the metabolic profile of diabetic children born to diabetic fathers and mothers.

## Conclusions

Familial T1D seems to have a less severe initial metabolic presentation than non-familial T1D, but this difference tends toward equality during follow-up. The risk of T1D for the offspring of an affected father is higher than for an affected mother. This still-mysterious preferential transmission was also confirmed in our study with a slightly better evolutionary profile when the mother has T1D. We recommend further studies in this area to explain the genetic and environmental mechanisms responsible for this divergence between the two parents.
